# Significantly different roles of economic affluence in sex-specific obesity prevalence rates: understanding more modifications within female body weight management

**DOI:** 10.1038/s41598-022-19633-3

**Published:** 2022-09-21

**Authors:** Wenpeng You, Maciej Henneberg

**Affiliations:** 1 School of Biomedicine and Adelaide Nursing School, The University of Adelaide, Adelaide, SA 5005 Australia; 2 Institute of Evolutionary Medicine, University of Zurich, Zurich, Switzerland; 3 Unit for Biocultural Variation in Obesity, University of Oxford, Oxford, UK

**Keywords:** Health care, Public health, Weight management

## Abstract

Socioeconomic status has been associated with obesity prevalence increase in both males and females worldwide. We examined the magnitude of the difference between the two relationships and explored the independence of both relationships. Country specific data on gross domestic product (GDP) per capita, sex-specific obesity prevalence rates, urbanisation, total calories availability and level of obesity, genetic background accumulation (measured by the Biological State Index, I_bs_) were obtained for 191 countries. Curvilinear regressions, bivariate and partial correlations, linear mixed models and multivariate linear regression analyses were used to examine the relationship between GDP and obesity prevalence rates in males and females respectively. Fisher’s r-to-z transformation, F-test and R^2^ increment in multivariate regression were used to compare results for males and females. GDP significantly correlated with sex-specific obesity prevalence rates, but significantly more strongly with male obesity prevalence in bivariate correlation analyses. These relationships remained independent of calories availability, I_bs_ and urbanization in partial correlation model. Stepwise multiple regression identified that GDP was a significant predictor of obesity prevalence in both sexes. Multivariate stepwise regression showed that, when adding GDP as an obesity prevalence predictor, the absolute increment of R^2^ in male fit model (0.046) was almost four (4) times greater than the absolute increment in female model fit (0.012). The Stepwise analyses also revealed that 68.0% of male but only 37.4% of female obesity prevalence rates were explained by the total contributing effects of GDP, I_bs_, urbanization and calories availability. In both Pearson’s r and nonparametric analyses, GDP contributes significantly more to male obesity than to female obesity in both developed and developing countries. GDP also determined the significant regional variation in male, but not female obesity prevalence. GDP may contribute to obesity prevalence significantly more in males than in females regardless of the confounding effects of I_bs_, urbanization and calories. This may suggest that aetiologies for female obesity are much more complex than for males and more confounders should be included in the future studies when data are available.

## Introduction

Obesity (defined by body mass index, BMI ≥ 30 kg/m^2^) is a complex, multifactorial, and largely preventable condition^1,2^. Enormous efforts have been made to control obesity increase all over the world. However, unfortunately, except Singapore who implemented very aggressive exercise based programmes^3^, no other country has achieved their expected results in the past decades^1^. Now, obesity, along with overweight, has been affecting over a third of the world’s population^1,2,4^. Obesity has been considered a major risk factor for various health issues^5–7^, such as cardiovascular diseases, type 2 diabetes, osteoarthritis, premature death, depression and certain types of cancer^8–10^.

The causes of obesity may be both biological and social^11^. However, research into biological factors has been far more thorough than into social risk factors, and biological research and the importance of its findings continue to grow. GDP has been significantly and strongly associated with total calories availability (r > 0.7, p < 0.001) in previous studies^12,13^. Accordingly individual major food groups which contribute to total calories availability have also been associated with obesity, such as fat^14–16^, meat protein^13,17,18^, carbohydrates^19,20^ and soybean^21^. Gluten in cereal crops and trypsin and phytates in soy food products have been associated with metabolic syndrome leading to obesity^12,21,22^. Urbanization and urban industries contribute to more than 80% of global GDP^23^, but it has been associated with sedentary lifestyle and poor diet structure, which have been posing major risk factors for obesity^24–29^. In the recent years, better healthcare services in developed countries have been thought to reduce natural selection and allow the accumulation of obesity related genetic background, which genetically contributes to population level obesity prevalence^30–32^.

Veblen in 1889 may be the first scientist to relate socioeconomic status (SES) to body weight in both sexes, males and females^33^. One hundred years later, a review of 144 published studies of the relationship between SES and obesity reveals a strong inverse relationship among women, but inconsistent for men in developed societies^34–37^. Contrarily, in developing countries, there are not enough studies on the relationships between SES and female and male obesity for drawing conclusions yet^35,38^. The underlying reasons may be that there have been no sufficient comparable data to explore and compare the sex-specific correlations for males and females at population level, for example between developed and developing countries, or within a nation.

Constantly, studies have revealed that obese people would have significant health benefit if they lose their weight moderately^39–41^. Due to physiological differences, males and females may have different negative consequences from obesity. For instance, obesity may be nearly three times more deadly for men than it is for women^42^. However, females are at higher risk of developing morbid obesity than males^43^.

With the advantage of ecological studies to have more access to population level information, this study aimed to supplement the previous studies with the internationally comparable data for exploring and comparing the correlations of GDP to sex-specific obesity prevalence rates. In order to examine the independent relationships, total calories availability, urbanization, and accumulation of genetic background of obesity (measured by Biological State Index, (I_bs_)) were included in the data analyses as the potential confounders.

## Materials and methods

The population (country) level data were obtained from international organisations for this ecological study.

### Data sources

Gross domestic product per capita (GDP, expressed in US dollars 2010) was extracted from the website of the World Bank as the independent variable^44^. It will be called “economic affluence” interchangeably in this study.

The WHO Global Health Observatory (GHO) data (2014) on estimated sex-specific obesity prevalence rates by country were obtained and used as the dependent variables^45^. The estimates of sex-specific prevalence rates of obesity are expressed as the percentages of population aged 18+ with BMI equal to or over 30 kg/m^2^.

There are 3 country specific potential confounding factors in this study. We intentionally backdated the years of predicting variable, GDP, and potential confounding variables, calories, obesity genetic background accumulation and urbanization, because of their delayed presentations in obesity prevalence. For example, males who moved today into urban areas, may not be obese the next day. It may take some years for their bodies to accumulate fat.

1. Calories availability, expressed as the mean grand total calories supply/availability per capita per day during the period of 3 years 2011–2013^46^.
  Overconsumption of calories due to increased affordability has been a well-established risk factor for body weight increase^47–49^. We need to acknowledge that calories availability published by the FAO only means the availability of food products which may not be the actual intake, although a large majority of the available food products is consumed by people.
2. Biological State Index (I_bs_), estimating the magnitude of accumulation of obesity genetic background and other obesity associated deleterious genes in a population due to relaxed natural selection^30,32,50,51^.
  I_bs_ calculation was based on the fertility data of each country published by United Nations^52–54^ and the mortality data of life tables published by World Health Organization (WHO) in 2012^55^. These calculations were the same as in the previous study published by Budnik and Henneberg^32^.
3. Urbanization (URBAN), expressed as a percentage of the population living in urban areas in 2010^56^
  Living in an urban setting leads to sedentary lifestyle (less physical activity) and poor diets (sugar, less vegetables), which have been considered an important factor to increase the risk of obesity^57–60^. Urban living setting also mirrors the Western lifestyle.

The independent variable (GDP) and all the three potential confounders were aligned with the listing of the prevalence rates of obesity (BMI ≥ 30 kg/m^2^) in females and males. A set comprising 191 country specific data was established and put in a uniform format in the Microsoft^®^ Excel 2016 for subsequent data analysis. Each country was treated as an individual subject and all of their available information was analysed. For some countries an estimate of one or the other variable was missing, thus specific analyses have sample sizes varying from 168 to 191.

International organizations, such as the WHO, FAO and the World Bank monitor and publish population specific data in relation to the health status, nutrition and diet, and economic development. These data have been helping governments, policymakers, funders and researchers track and investigate the priorities of health research and development based on public health needs and ensure that funds and resources are used to meet the priorities. Their data have been recently used to examine the relationships between nutrients and obesity^12,13,21,61,62^, diabetes^51,63–65^, and relationship between natural selection and obesity^32,66^ and type 1 diabetes^51^ and cancers^66–68^ respectively.

Data are freely available from the websites of the UN agencies (WHO, the World Bank and FAO). Data sources were described in the manuscript and their specific URLs were indicated in the section of References. No ethical approval or written informed consent for participation was required.

### Data multicollinearity check

We conducted the diagnostic test to rule out the potential multicollinearity between the variables for our data analyses. The test criteria are set up with the tolerances over 0.20 and the Variance Inflation Factors (VIF) less than 5^69^.

### Data analysis

The country specific data were collected for the ecological analyses in this study, which proceeds with six steps:

1. Microsoft Excel^®^ was applied to produce scatter plots with raw (non-logarithmed) data for exploring and visualizing the worldwide relationships between the GDP and the obesity prevalence rates in males and females respectively. Accordingly, the two trendlines were indicated in the graphs to reflect the relationships between economic affluence and obesity prevalence rates in males and females respectively.
  For other analyses in SPSS, the variable values were logarithmically transformed to bring their distributions closer to normality. This allowed us to examine and compare the correlations between economic affluence and obesity prevalence rates in males and females worldwide, and in different country groupings.
2. The Pearson product-moment correlation coefficients (Pearson’s r) were explored for the measures of the strengths of a linear association between variables (independent, dependent and potential confounders).
  Considering the deviations from homoscedasticity, subsequently, nonparametric correlation (Spearman’s rho) analysis was performed with the same set of data to examine the magnitude of the potential differences between correlation coefficients for each sex-specific obesity prevalence rate and all variables calculated in Pearson and nonparametric correlation analyses.
3. Partial correlation analysis was performed to explore the independent linear correlations of GDP to male and female obesity prevalence rates respectively while we controlled for calories availability, I_bs_ and urbanization.
  Fisher’s r-to-z transformation was conducted to assess the level of difference and its significance in each individual association between GDP and sex-specific obesity prevalence rate in the data analysis models of Pearson’s r, Spearman’s rho and partial correlation.
4. Standard multivariate linear regression (Stepwise) was performed to assess which non-GDP predictor(s) made substantial contributions to variation in obesity, and then GDP was added to the list of predictors to show improvement in model fits for males and females. The magnitudes of improvements in the two model fits were compared with the absolute improvement values obtained from “the R^2^ improvement in male prevalence due to adding GDP” and “the R^2^ improvement in female prevalence due to adding GDP” respectively.
  Standard multivariate linear regression (Enter) was conducted on log-transformed data to obtain and compare the Beta coefficients between sex-specific obesity prevalence and all independent variables, which included economic affluence, calories availability, I_bs_ and urbanisation. The two Beta coefficients were compared to test the level of difference^70^.
5. The universal correlations between GDP and sex-specific obesity prevalence rates were explored and compared (Fisher’s r-to-z transformation) in different country groupings:
  1. the UN common practice of defining the developed and developing countries^71^;
  2. the World Bank income classifications: high income, upper middle income, low-middle income and low income;
  3. the WHO regional classifications: Africa (AFR), Americas (AMR), Eastern Mediterranean (EMR), Europe (EU), South-East Asia (SEAR) and Western Pacific (WPR)^72^;
  4. countries with the strong contrast in terms of geographic distributions, per capita GDP levels and/or cultural backgrounds. We analysed the correlation in the six country groupings: Asia Cooperation Dialogue (ACD)^73^; The Asia–Pacific Economic Cooperation (APEC); the Arab World^74^, countries with English as the official language (government websites), European Economic Area (EEA)^75^, European Union (EU)^76^, Latin America and the Caribbean (LAC)^77^, the Organisation for Economic Co-operation and Development (OECD) and non-OECD grouping^78^, and the Southern African Development Community (SADC)^79^.
  In our analysis, we only included those countries for which we could access their data for the specific groupings. To a large extent, grouping countries for analysis may also allow to align our findings against previous local or regional studies regarding heterogeneous epidemiology approaches due to various geographic locations and ethnicity.
6. The importance of GDP in determining the regional variation of sex-specific obesity prevalence rates was compared within the six (6) WHO regions, and UN developed and developing country groupings respectively.

Obesity prevalence rates in both males and females vary by region significantly and the variations have rapidly increased between 1980 and 2008^11,80^. The role of GDP in determining significant regional variations of obesity prevalence rates in males, but not in females, between the six (6) WHO regions was demonstrated.

Equations of the best fitting trendline displayed in the scatter plots analysis of relationships between GDP and obesity prevalence rates in males and females were used to calculate and remove the contributing effect of GDP on obesity prevalence rate in males and females respectively. This allowed us to create two new dependent variables, “Residual of male obesity prevalence standardised on GDP” and “Residual of female obesity prevalence standardised on GDP”. Countries were categorized as per the WHO regions^72^ and UN common practice on defining more developed and developing countries^71^ for investigating the regional variations based on mean difference. Means of obesity prevalence rates in both males and females, and “Residual of male obesity prevalence standardised on GDP” and “Residual of female obesity prevalence standardised on GDP” of all the countries were calculated for each of the six WHO regions, and UN developed and developing countries. Post hoc Scheffe (Oneway ANOVA) and Independent Samples T-test were conducted to compare the means between the six WHO regions and UN country groupings respectively.

Standard deviation is a measure of how dispersed the data are in relation to the mean. Low standard deviation means data are clustered around the mean, and high standard deviation indicates data are more spread out. We calculated the sex-specific standard deviations used for facilitating our Discussion.

Pearson’s r, Spearman’s rho coefficient, partial correlation, the linear Mixed Model Analysis and multiple-linear regression analyses were conducted using SPSS v. 25. The statistical significance was set at the 0.05 level, but the significance levels at 0.01 and 0.001 were also reported.

## Results

**Figure 1:**
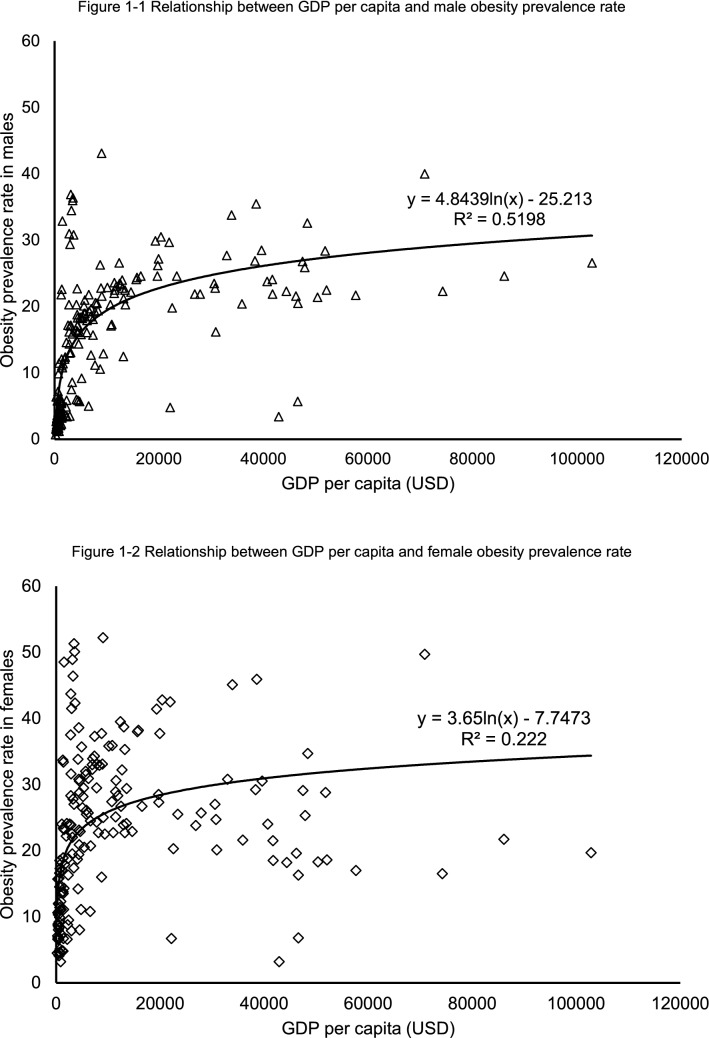
Relationship between GDP per capita and sex-specific obesity prevalence rate.

Economic affluence (GDP) was in strong and significant correlation with both male obesity prevalence rate (logarithmic, r = 0.721 p < 0.001, Fig. 1–1) and female obesity (logarithmic, r = 0.471, p < 0.001, Fig. 1–2). Fisher r-to-z revealed that GDP was in significantly stronger correlation with male obesity than with female obesity (z = 3.21, p < 0.001). GDP explained 51.98% of male obesity prevalence variance, which is more than double female obesity prevalence variance (22.20%).

In Pearson correlation analysis, worldwide, GDP was significantly correlated with both male and female obesity prevalence rates (Table 1). Similar values of correlation coefficients were observed in Spearman’s rho analysis as well indicating that log-transformation is sufficient to avoid substantial deviations from linear regressions in moment-product correlations (Table 1).

**Table 1:** Pearson r correlation (above the diagonal) and Spearman rho (below the diagonal) between all variables.

	GDP per capita	BMI ≥ 30, 18+ male	BMI ≥ 30, 18+ female	Calories availability	Urbanization	I_bs_
GDP	1	0.761***	0.517***	0.759***	0.672***	0.710***
BMI ≥ 30, 18+ male	0.758***	1	0.903***	0.716***	0.580***	0.692***
BMI ≥ 30, 18+ female	0.504***	0.845***	1	0.493***	0.399***	0.470***
Calories availability	0.756***	0.742***	0.451***	1	0.602***	0.639***
Urbanization	0.736***	0.583***	0.372***	0.660***	1	0.666***
I_bs_	0.866***	0.667***	0.371***	0.765***	0.736***	1

Pearson (two-tailed) is reported. Number of countries included in the analysis range from 172 to 191.

Data sources: Total calories availability data from the FAO’s FAOSTAT. BMI ≥ 30 prevalence (male and female) from the WHO Global Health Observatory; GDP per capita from the World Bank; Urbanization data from WHO; I_bs_ from the previous publications.

***All correlations are significant at the 0.001 level (two-tailed).

Fisher r-to-z transformation revealed that GDP significantly more strongly correlated with male obesity than with female obesity in Pearson’s r (z = 4.06, p < 0.001) and Nonparametric correlation (z = 4.16, p < 0.001) respectively (Table 2).

**Table 2:** Correlation coefficients and Fisher’s r-to-z transformations of bivariate and partial correlations between GDP per capita and female and male obesity prevalence.

Variable	Pearson correlation GDP				Nonparametric correlation GDP				Partial correlation GDP				
	n	r	p	Fisher's r-to-z transformation	N	r	P	Fisher's r-to-z transformation	df	r	p	Effect Size	Fisher's r-to-z transformation
BMI 30, M	184	0.761	< 0.001	z = 4.06 p < 0.001	184	0.758	< 0.001	z = 4.16 p < 0.001	163	0.332	< 0.001	0.110	z = 1.64 p < 0.05
BMI 30, F	184	0.517	< 0.001		184	0.504	< 0.001		163	0.160	< 0.05	0.026	
Calories availability	168	0.759	< 0.001	–	168	0.756	< 0.001	–	–	–	–	–	–
Urbanization	184	0.672	< 0.001	–	184	0.736	< 0.001	–	–	–	–	–	–
I_bs_	184	0.710	< 0.001	–	184	0.866	< 0.001	–	–	–	–	–	–

Bivariate and partial correlations are reported. –, Controlled variable or not relevant.

Data sources: Total calories availability data from the FAO’s FAOSTAT. BMI ≥ 30 prevalence (male and female) from the WHO Global Health Observatory; GDP per capita from the World Bank; Urbanization data from WHO; I_bs_ from the previous publications.

Partial correlation analysis showed that, worldwide, the GDP was still significantly correlated with the male and female obesity prevalence while we controlled for calories availability, I_bs_ and urbanization (Table 2). GDP was in partial correlation significantly stronger correlated with male obesity prevalence than with female obesity prevalence (z = 1.64, p < 0.05) (Table 2).

The effect size of GDP on male obesity prevalence is 0.110, which is much greater than on female prevalence, 0.026 (Table 2).

The above results suggest that economic affluence indexed with GDP per capita played a significantly stronger role in male obesity than in female obesity.

Multivariate regression model (Enter) revealed that GDP was a strongly significant predictor of male obesity prevalence when I_bs_, calories availability, GDP and urbanization were entered as the predicting variables. In contrast, GDP was only a relatively weak, though still significant predictor of female obesity prevalence (Table 3–1). The influence of GDP on male obesity prevalence rate was stronger than on female obesity prevalence (overlap = 0.033).

**Table 3:** Results of linear regression analyses to describe the relationships between obesity prevalence rates and their predictors in females and males respectively.

1. Enter model													
Variable	Male obesity prevalence					Female obesity prevalence							
	GDP excluded		GDP included			GDP excluded			GDP included				
	Beta	Sig	Beta	Sig	SE	Variable	Beta	Sig	Beta	Sig	SE		
GDP	–	–	0.360	< 0.001	0.050	GDP	–	–	0.247	< 0.05	0.048		
Calories availability	0.354	< 0.001	0.175	< 0.05	0.409	Calories availability	0.266	< 0.01	0.095	0.366	0.396		
I_bs_	0.376	< 0.001	0.287	< 0.001	0.638	I_bs_	0.222	< 0.05	0.180	0.060	0.618		
URBAN	0.202	< 0.001	0.126	< 0.05	0.113	URBAN	0.142	0.090	0.112	0.212	0.110		

2. Stepwise													
Model	Male obesity prevalence						Female obesity prevalence						
	GDP excluded		GDP included				GDP excluded			GDP included			
	Variable	Adjusted R^2^	Variable	Adjusted R^2^	Beta	SE	Model	Variable	Adjusted R^2^	Variable	Adjusted R^2^	Beta	SE
1	Calories availability	0.509	GDP	0.606	0.360	0.050	1	Calories availability	0.239	GDP	0.268	0.521	0.027
2	I_bs_	0.611	I_bs_	0.657	0.287	0.638	2	I_bs_	0.272	I_bs_	0.284	0.374	0.038
3	URBAN	0.634	Calories	0.673	0.175	0.409	3	URBAN	Insig.	Calories availability	Insig	–	–
4	–		URBAN	0.680	0.126	0.113	4	–	–	URBAN	Insig	–	–

Enter and Stepwise multiple linear regression modelling are reported.

Data sources: Total calories data from the FAO’s FAOSTAT. BMI ≥ 30 prevalence (male and female) from the WHO Global Health Observatory; GDP per capita from the World Bank; Urbanization data from WHO; I_bs_ from the previous publications.

SE: standard error; Insig.: insignificant.

Stepwise multivariate regression model results indicated that GDP was strongest and significant predictor of both male and female obesity prevalence. However, GDP explained 60.6% male obesity prevalence, but only 26.8% female obesity prevalence (Table 3–2). The influence of GDP on male obesity prevalence rate was significantly stronger than on female obesity prevalence (overlap = 0.00%).

In the Stepwise regression, the absolute improvement of R^2^ value due to adding GDP in male model fit was 0.046 (from 0.634 to 0.680), which was nearly four times the absolute improvement value 0.012 (from 0.272 to 0.284) due to adding GDP to female model fit (Table 3–2).

In the Stepwise multivariate regression model, when calories availability, GDP, I_bs_ and urbanization were included as the independent predicting variables, all the four variables, explaining 68.0% male obesity in total, were selected as the predictors that have the most influence on male obesity (Table 3–2). Interestingly, only GDP and I_bs_ were selected as the predictor which have the most influence on female obesity, and all the four variables only explain 28.4% female obesity in total (Table 3–2). This may suggest that, statistically, female obesity can be explained by other factors, such as psychological and social expectations etc*.*

Table 4 shows and compares the bivariate relationships between GDP and male and female obesity prevalence rates in different country groupings. The general trend was that, in the wealthy countries, GDP correlated with male obesity prevalence rate significantly stronger than it correlated with female obesity prevalence rate. This can be observed in the UN developed countries, the World Bank high income countries, the WHO Europe regional area, European Economic Area, European Union and Organisation for Economic Co-operation and Development. Contrarily, in the countries with lower GDPs, differences between GDP correlations with male and female obesity prevalence rates were generally smaller and insignificant.

**Table 4:** Comparisions of bivariate ccorrelation of GDP to sex-specific obesity prevalence rates in different country groupings.

Country groupings	Pearson			Nonparametric		
	Male	Female	Fisher's r-to-z transformation	Male	Female	Fisher's r-to-z transformation
Worldwide, n = 184	0.761***	0.517***	z = 4.06, p < 0.001	0.758***	0.504***	z = 4.16, p < 0.001
**UN developed and developing country groupings**						
Developed countries, n = 44	0.270	− 0.115	z = 1.780, p = 0.0375	0.506***	− 0.165	z = 3.28, p = 0.0005
Developing countries, n = 140	0.772***	0.648***	z = 2.100, p = 0.0179	0.769***	0.672***	z = 1.68, p = 0.0465
**World Bank income classifications**						
Low income, n = 30	0.645***	0.548**	z = 0.560, p = 0.2877	0.580***	0.558**	z = 0.120, p = 0.4522
Low middle income, n = 49	0.541***	0.4920***	z = 0.320, p = 0.3745	0.574***	0.560***	z = 0.100, p = 0.4602
Upper middle income, n = 52	0.099	0.100	z = 0.000, p = 0.5000	0.217	0.124	z = 0.470, p = 0.3192
High income, n = 53	0.006	− 0.308*	z = 1.620, p = 0.0526	0.093	− 0.418**	z = 2.690, p = 0.0036
**WHO regions**						
Africa (AFR), n = 46	0.872***	0.847***	z = 0.440, p = 0.3300	0.834***	0.847***	z = − 0.210, p = 0.4168
Americas (AMR), n = 35	0.954***	0.697***	z = 4.506, p = 0.0000	0.944***	0.658***	z = 3.940, p = 0.0000
Eastern Mediterranean (EMR), n = 19	0.877***	0.849***	z = 0.310, p = 0.3783	0.960***	0.936***	z = 0.680, p = 0.2483
Europe (EUR), n = 51	0.829***	0.697	z = 5.92, p = 0.0000	0.699***	− 0.051	z = 4.492, p = 0.0000
South-East Asia (SEAR), n = 9	0.867**	0.861**	z = 0.040, p = 0.4840	0.883**	0.883**	z = 0.000, p = 0.5000
Western Pacific (WPR), n = 24	0.080	− 0.102	z = 0.590, p = 0.2776	0.172	0.049	z = 0.400, p = 0.3446
**Countries grouped based on various factors**						
Asia Cooperation Dialogue (ACD), n = 32	0.653***	0.707***	z = − 0.380, p = 0.3520	0.651***	0.702***	z = − 0.360, p = 0.3594
Asia–Pacific Economic Cooperation (APEC), n = 19	0.425*	0.208	z = 0.690, p = 0.2451	0.551*	0.284	z = 0.930, p = 0.9300
Arab World (AW), n = 18	0.877***	0.860***	z = 0.190, p = 0.4247	0.963***	0.942***	z = 0.630, p = 0.2643
Countries with English as official language (EOL), n = 53	0.700***	0.496***	z = 1.620, p = 0.0526	0.658***	0.492***	z = 1.250, p = 0.1056
European Economic Area (EEA), n = 30	0.293	− 0.482**	z = 3.040, p = 0.0012	0.180	− 0.473***	z = 2.300, p = 0.0107
European Union (EU), n = 28	0.260	− 0.477**	z = 2.780, p = 0.0027	0.110	− 0.417*	z = 1.960, p = 0.0250
Latin America Caribbean (LAC), n = 33	0.960***	0.778***	z = 3.510, p = 0.0002	0.933***	0.689***	z = 3.230, p = 0.0006
Organisation for Economic Co-operation and Development (OECD), n = 34	0.043	− 0.274	z = 1.280, p = 0.1003	0.085	− 0.447**	z = 2.230, p = 0.0129
Southern African Development Community (SADC), n = 15	0.983***	0.886***	z = 2.309, p = 0.0084	0.974***	0.896***	z = 1.705, p = 0.0401

*p < 0.05, **p < 0.01; ***p < 0.001; Data sources: Total calories data from the FAO’s FAOSTAT. BMI ≥ 30 prevalence (male and female) from the WHO Global Health Observatory; GDP per capita from the World Bank; Urbanization data from WHO; I_bs_ from the previous publications.

The significant difference was also observed in the different country groupings with stratified socioeconomic levels. For instance, the difference between male and female obesity correlations with GDP were greater and significant in UN developing country grouping while not in the developed countries respectively. The similar pattern occurred between the low-income countries and the high-income countries in the World Bank country classifications.

Table 5 shows that GDP determined regional variation of male obesity prevalence rate, but not female obesity prevalence rate. Post hoc Scheffe analysis of 30 comparisons of means between the six (6) WHO regions, found in 18 out of 30 the significant differences in male obesity prevalence rate. However, all the eighteen (18) differences lost the significant levels when the contribution to obesity prevalence of GDP was removed in the same analysis model. The same analysis approach was applied to compare the means of female obesity prevalence rate. With and without GDP contributions to female obesity prevalence rate, the numbers of significant differences remained the same within the six (6) WHO regions.

**Table 5:** Comparisons between sex-specific obesity prevalence rates between WHO regions, and between UN developed and developing regions.

	Male obesity		Male obesity residual standardised on GDP		Female obesity		Female obesity residual standardised on GDP	
Post hoc Scheffe, WHO regions								
I (region)	J (region)	Mean difference (I-J)	J (region)	Mean difference (I-J)	J (region)	Mean difference (I-J)	J (region)	Mean difference (I-J)
AFRO, n = 46	AM	− 13.49***	AM	− 5.14	AM	− 14.55***	AM	− 7.63*
	EM	− 14.22***	EM	− 6.19	EM	− 14.89***	EM	− 9.38*
	EU	− 15.60***	EU	0.17	EU	− 7.45*	EU	3.38
	SEA	2.34	SEA	3.22	SEA	8.61	SEA	9.49
	WP	− 17.04***	WP	− 6.84	WP	− 14.48***	WP	− 5.80
AMRO, n = 35	AF	13.49***	AF	5.14	AF	14.55***	AF	7.63*
	EM	− 0.73	EM	− 1.05	EM	− 0.34	EM	− 1.76
	EU	− 2.11	EU	5.31	EU	7.10	EU	11.00***
	SEA	15.83***	SEA	8.36	SEA	23.16***	SEA	17.10***
	WP	− 3.55	WP	− 1.70	WP	0.07	WP	1.82
EMRO, n = 19	AF	14.22***	AF	6.19	AF	14.89***	AF	9.38*
	AM	0.73	AM	1.05	AM	0.34	AM	1.76
	EU	− 1.38	EU	6.36	EU	7.45	EU	12.76***
	SEA	16.56***	SEA	9.41	SEA	23.51***	SEA	18.86*
	WP	− 2.83	WP	− 0.65	WP	0.41	WP	3.58
EURO, n = 51	AF	15.60***	AF	− 0.17	AF	7.45*	AF	− 3.38
	AM	2.11	AM	− 5.31	AM	− 7.10	AM	− 11.00***
	EM	1.38	EM	− 6.36	EM	− 7.45	EM	− 12.76***
	SEA	17.94***	SEA	3.05	SEA	16.06***	SEA	6.11
	WP	− 1.44	WP	− 7.01	WP	− 7.03	WP	− 9.17*
SEARO, n = 9	AF	− 2.34	AF	− 3.22	AF	− 8.61	AF	− 9.49
	AM	− 15.83***	AM	− 8.36	AM	− 23.16***	AM	− 17.10***
	EM	− 16.56***	EM	− 9.41	EM	− 23.51***	EM	− 18.86***
	EU	− 17.94***	EU	− 3.05	EU	− 16.061	EU	− 6.11
	WP	− 19.39***	WP	− 10.06	WP	− 23.09***	WP	− 15.28**
WPRO, n = 24	AF	17.04***	AF	6.84	AF	14.48***	AF	5.80
	AM	3.55	AM	1.70	AM	− 0.07	AM	− 1.82
	EM	2.83	EM	0.65	EM	− 0.41	EM	− 3.58
	EU	1.44	EU	7.01	EU	7.03	EU	9.17*
	SEA	19.39***	SEA	10.06	SEA	23.09***	SEA	15.28**

Independent T-test								
	With GDP contributions		T	Obesity rate residuals standardised on GDP		T		
United Nations region classifications based on common practice	Developed (n = 44) vs. Developing (n = 140)	Male obesity prevalence	4.549***	Developed (n = 44) vs. Developing (n = 140)	Male obesity prevalence	4.325***		
		Female obesity prevalence	0.388		Female obesity prevalence	5.551***		

Data sources: Total calories data from the FAO’s FAOSTAT. BMI ≥ 30 prevalence (male and female) from the WHO Global Health Observatory; GDP per capita from the World Bank; Urbanization data from WHO; I_bs_ from the previous publications.

AF, Africa; AM, Americas; EM, Eastern Mediterranean; EU, Europe; SEA, South-East Asia; WP, Western Pacific; RO, Regional Office.

*p < 0.05, **p < 0.01; ***p < 0.001.

This was further confirmed with the Independent T-test to compare the sex-specific obesity prevalence rates in the UN developed and developing countries. The mean of male obesity prevalence rate was significantly different between developed and developing country groupings (T = 4.549, p < 0.001). However, for female obesity prevalence rate, the difference was negligible and insignificant (T = − 0.388) between developed and developing country groupings. When GDP contributions to male and female obesity prevalence rates were removed, the mean of male obesity prevalence rate in developed country grouping is significantly lower than that in developing country grouping (T = 4.33, p < 0.001). However, when GDP contributions were removed, the mean differences of prevalence rates in males and females between developed and developing countries are similar (T = 5.551 vs 4.325, p < 0.001).

## Discussion

The findings in this study confirmed that both male and female obesity prevalence rates were in non-linear relationships with GDP per capita as in previous studies. The scatter plots showed that sex-specific obesity prevalence rates initially increase with income, flatten out and then are attenuated with further GDP increase. However, the difference between the associations of GDP with sex-specific obesity prevalence rates is more complex than often argued.

With the advantages of ecological studies, we extracted the comparable data for examining and comparing the correlations between GDP and obesity prevalence rates in males and females globally and in different country clusters. Our data analyses revealed that, worldwide, GDP per capita was in significantly stronger correlations with male obesity prevalence rates than with female obesity prevalence rates. Furthermore, this significantly different relationship was independent of other major obesity risk factors, total calories availability, urban lifestyle and obesity genetic background accumulation. We also found that, in developing countries, the correlations of both sex-specific obesity prevalence rates with GDP were stronger than their counterparts in the developed countries.

Worldwide, GDP has been a major drive for obesity prevalence rate. However, surprisingly, it has fuelled male obesity prevalence rate significantly more than it has done for female obesity prevalence rate. It is not clear to us how genetic background influences the correlations between GDP and sex-specific obesity prevalence rate. There are several possible environmental factors which may have been altering correlations of GDP with obesity prevalence rates more in females than males^30^. Males and females may be differently exposed to socioeconomic inequalities. Wells et al*.* argued that in developing countries, females with low and insecure income may not have as much access to nutritious food as males, and this may influence their body mass increase^81^. Worldwide, different sociocultural beliefs and practices may affect female disparities in excessive weight gain^11,82–86^. Discriminatory social practices measured with the Gender Inequality Index, the Global Gender Gap Index and the Social Institutions and Gender Index were applied to explain the more prevalent female obesity^87^.

An important double social standard on people’s appearance has made females more appearance-focused than males^88–90^ which leads females to modify their personal environment, such as work routine, dietary pattern and physical exercise to adjust their body mass to appearance standards^90,91^. Therefore, although females may share with males the same level of natural genetic endowment for the phonotype of body weight, their body weights have been influenced by specific practices to meet different social and cultural values. For instance, in the developing world, secularly, a large body size of female is desired and considered a sign of wealth and health^92,93^, which is even linked to higher fertility^11^ as reproduction is a nutritionally expensive process for women^94,95^. Additionally, in some cultures and religions^96^, females have been overprotected and restricted from publicly participating in physical activities^97^. These can be seen in not only some developing countries, for instance, North Africa^98^ and Middle East^99^ regions, but also in some developed countries such as Saudi Arabia^100^, Kuwait^101,102^ and Oman^101,103^.

In the economically transitioning countries, agriculture industrialisation has replaced more agrarian laborious jobs of females than males. Accordingly, this transition has reduced the physical activity for females more than males which may have increased the obesity prevalence rates in females more than in males^11,104,105^. Agriculture industrialisation toward the end of the twentieth century also released more females to become salaried workforce^106,107^. For time-saving purposes, they may choose to purchase more pre-packaged foods leading to body weight increase^108^. Those females who experienced periods of deprivation during childhood, may purchase more food due to their stronger motivation to ensure food security^109^.

In contrast, in developed countries, lots of females practice more healthy lifestyles, such as healthy diet patterns^110,111^ and more physical exercise^112,113^ and even psychological meditation^114–116^. These body control approaches may have worked successfully which is typically reflected with the inverse correlations between GDP and female obesity prevalence rates in our bivariate correlation models (Table 4). However, in the Western societies, females still show greater obesity prevalence than those in developing countries. This may have several explanations. Females in the developed world show greater consumption of those foods and drinks which pose higher risk for gaining body weight, such as alcoholic beverages^117,118^, sugar^62,119^, and meat products^13,62,120^. Social network is culturally more acceptable in the Western world for females, and it may exacerbate their trend to gain more body weight^82,121^. Additionally, their declining total fertility rate leading to the increase of estrogen production may explain their body weight increase as well^122–124^. At population level, it is reflected with greater obesity prevalence rates.

The above factors have modified the population level male and female obesity prevalence significantly. To support this statement, we compared the calculated standard deviations of male and female obesity prevalence rates (10.5182 and 12.1482 respectively) with the F-test. It was identified that there is a significant difference between the two standard deviations (F = 1.33, p < 0.05). This may indicate that female obesity prevalence rates in different countries are more spread due to more complex modifications, which include those above mentioned factors.

Greater economic affluence may allow people to have more opportunity to modify body weight though a number of interventions, which may be significant depending on socioeconomic status. Driven more by appearance, females may take more advantage of greater economic affluence to manage their body weight. The scatterplots exploring the relationships between male and female obesity prevalence show that, in the developed countries, male obesity prevalence only explains 59.51% of female obesity, but 92.14% in the developing countries. This may be partially because females in the developed countries have been exerting more modifications to manage their body weight.

The similar sources of data on economic affluence and sex-specific obesity prevalence rates were extracted and their relationships have been examined by other researchers. Wells et al. reported the correlations of GDP to obesity prevalence rates in both males and females at population level^81^. However, in their study the correlation of GDP to female obesity prevalence rates did not reach the significant level. Most likely, this was due to the small sample size (n = 73, instead of 191 with available data on obesity prevalence rates). This may have led to biased correlation which could not represent the general worldwide correlations between GDP and sex-specific obesity prevalence rates. The other significant difference from our study is that the potential major confounders were not appropriately considered. Total fertility rate was included in the regression model for exploring that GDP correlated with female obesity prevalence rate independently, but it has not been established as a possible risk factor for obesity yet. Our study included the three well established population level potential confounders of obesity (calories availability, I_bs_ and urbanization), and we found that the GDP correlated with obesity prevalence rates in both sexes significantly and independently in both partial correlation and multivariate linear regression models. With large similarities, the non-linear relationships between GDP and sex-specific obesity prevalence rates were observed and economic affluence variables were stratified for correlation analyses as well^125,126^. However, these studies did not quantify the level of differences in the correlation of GDP with sex-specific obesity prevalence rates. Nor did they include the potential confounders for GDP for ruling out their contributions to explore the independent correlations between GDP and sex-specific obesity prevalence rates. For instance, Eggers et al. acknowledged urban living as a major risk factor for obesity and analysed the relationships between urbanization and the obesity prevalence rates in both males and females^125^. However, the independence of the relationships was not considered in their study. At individual level, a study conducted in Korean population (45+) revealed that household income significantly correlated with male overweight, but not with female overweight prevalence due to cultural perception that drove females to manage their body weight for good looking body shape to increase their confidence and efficiency in work environment^90^. Obviously, high income applied more to male obesity increase than to females’ in the studied population. Again, the level of difference was not reported and the correlations of household income with sex-specific obesity were not corrected for any potential confounders.

Multiple variables (GDP, calories availability, urbanization and obesity gene accumulation) were included for the data analyses in this study. It is necessary to align our study findings with other related hypotheses.

High calories intake has been unarguably considered as the risk factor for obesity. In our study, the same total calories availability was correlated with both male and female obesity prevalence rates, but it significantly correlated with male obesity rather than with female obesity (z = 3.30, p < 0.001, and z = 4.31, p < 0.001) in Pearson’s and non-parametric correlations respectively, Table 1). This difference was confirmed in the subsequent Stepwise linear regression that calories availability was selected as the 3rd most influential predictor for male obesity (increasing R^2^ up to 0.673), but it was not shown as one of the most significant risk factors for female obesity prevalence (Table 3–2).

There are several limitations in this study. Firstly, like other data analysis-based studies, the relationships observed between the various variables may not be causal, but just correlational. Secondly, as this is an ecological study, with the intrinsic ecological fallacy, we could only demonstrate the relationships between the GDP and the sex-specific obesity prevalence rates at country/population level, which do not necessarily hold true at the individual level. Thirdly, Sex-specific obesity prevalence rates were not correlated with sex-specific exposures in this study due to data unavailability. However, it would be very interesting to see how the sex-specific obesity prevalences correlate to the sex specific exposure, such as economic welfare. Fourthly, the cross-sectional data, instead of longitudinal data, were analysed in this study. Therefore, the correlations identified may not be able to necessarily reflect historical trends of the relationships between economic affluence and sex-specific obesity prevalence rates. Finally, we controlled for the major risk factors involved in total calories availability, obesity genetic background accumulation (measured by I_bs_) and lifestyle change (measured by urbanization) in our data analyses, but there are still residuals of obesity which were not explained by these confounders and the independent variable. For instance, GDP and its three potential confounders explained 68.0% of male obesity prevalence rate, but only 28.4% of female obesity prevalence rate (Table 3–2). This also highlighted that more complex aetiologies of female obesity, such as adaptation for fertility^127^, oestrogen^124^ and double X chromosomes in cells^128,129^ may be at work. These factors may have confounded the correlations between GDP and sex-specific obesity prevalence rates, but we could not access or include the data for our analyses.

It is worth highlighting a strength or novelty of this study in comparison with the previous research in exploring the relationships between economic affluence and sex-specific obesity prevalence rates. The results in this study revealed that, surprisingly, the difference between females and males has reached the level of statistical significance globally. Furthermore, this significance level remains in different data analysis models with and without considering the competing effects of calories, urban lifestyles and genetic background of obesity accumulation.

## Conclusions

Worldwide, economic affluence (measured by GDP) is still the major drive for the increase of obesity prevalence rates in both sexes, but it plays a much stronger role in male obesity prevalence increase. Different from previous studies, we demonstrate that sexual disparity has reached the significance level, and is independent of the other common obesity drives, urbanization, calories availability and obesity associated genetic background.

## Supplementary Information

Supplementary Information.

## Data Availability

All data for this study are publicly available from the United Agencies’ websites. The specific sources are described in the section of “Data sources”, and a whole set of data for this study is attached as s Supplemental Excel document. The purpose of using these in this study meets the terms and conditions of the relevant UN agencies. The formal permission is not required to download and analyse the data in this study.
